# “WE’RE THE TARGET”: THE IMPACT OF CYBERCRIME AMONG OLDER ADULTS IN GHANA

**DOI:** 10.1093/geroni/igae098.2919

**Published:** 2024-12-31

**Authors:** Akwasi Adjei Gyimah, Eric Frimpong, Mathias Adjei

**Affiliations:** Miami University, Oxford, Ohio, United States; University of Massachusetts Boston, Boston, Massachusetts, United States; Miami University, Oxford, Ohio, United States

## Abstract

Among Older adults, the impact of experiencing cybercrime affects greatly on their quality of lives. Whilst maximum attention is directed towards issues relating to aging and experiences of cybercrime in the Western world, there is insufficient attention in the academic literature in Africa. This study explores the emotional and personal constraints endured by older adults in Ghana who have been victims of cybercrime. This study will serve to fill an important gap in knowledge and aid in the development of effective strategies to address the issue. Using a purposive criterion sampling and a semi-structured interview, 16 older adults aged 60 years and older who had reported cases of cybercrimes to the Asokwa Police Service Division, Kumasi-Ghana were recruited. An interpretative phenomenological analysis (IPA) was used. Older adults who are cyber-crime victims experience feelings of anger, frustration, violation and sometimes lead to them being depressed. Additionally, financial losses resulting from cybercrime significantly causes distress and financial difficulties to continue to age successfully. Especially, older adults who worked in the informal sector, have no social security returns to benefit from but only depends on their savings. Generally, respondents held divergent views on the effectiveness of civil and criminal justice systems in combating this phenomenon in Ghana. Interventions to curb cybercrimes are fundamentally significant. Especially, when most older people are at increased risk. There is the need for education and effective training on cyber security for older people and their caregivers.

